# Olfaction across the water–air interface in anuran amphibians

**DOI:** 10.1007/s00441-020-03377-5

**Published:** 2021-01-26

**Authors:** Lukas Weiss, Ivan Manzini, Thomas Hassenklöver

**Affiliations:** Institute of Animal Physiology, Department of Animal Physiology and Molecular Biomedicine, Justus-Liebig-University Giessen, Heinrich-Buff-Ring 38, 35392 Giessen, Germany

**Keywords:** Anura, Frog, Nose, Olfactory organ, Olfactory bulb, Odor mapping, Transduction, Olfactory epithelium, Vomeronasal organ, Olfactory cortex, Higher centers, Olfactory subsystems

## Abstract

Extant anuran amphibians originate from an evolutionary intersection eventually leading to fully terrestrial tetrapods. In many ways, they have to deal with exposure to both terrestrial and aquatic environments: (i) phylogenetically, as derivatives of the first tetrapod group that conquered the terrestrial environment in evolution; (ii) ontogenetically, with a development that includes aquatic and terrestrial stages connected via metamorphic remodeling; and (iii) individually, with common changes in habitat during the life cycle. Our knowledge about the structural organization and function of the amphibian olfactory system and its relevance still lags behind findings on mammals. It is a formidable challenge to reveal underlying general principles of circuity-related, cellular, and molecular properties that are beneficial for an optimized sense of smell in water and air. Recent findings in structural organization coupled with behavioral observations could help to understand the importance of the sense of smell in this evolutionarily important animal group. We describe the structure of the peripheral olfactory organ, the olfactory bulb, and higher olfactory centers on a tissue, cellular, and molecular levels. Differences and similarities between the olfactory systems of anurans and other vertebrates are reviewed. Special emphasis lies on adaptations that are connected to the distinct demands of olfaction in water and air environment. These particular adaptations are discussed in light of evolutionary trends, ontogenetic development, and ecological demands.

## Historical perspective

In scientific research, amphibians have a long history of use as a model to reveal basic principles of physiology, including the sense of smell (Burggren and Warburton 2007). The anatomy of the olfactory organs of amphibians has been investigated from the mid-nineteenth century when rich descriptions with highly detailed illustrations of their internal morphology became available (Ecker 1864–1882). Extensive reports about the structure of both the cellular components of the olfactory epithelium (OE) and the connection to the anterior telencephalon are available from early investigations (Schultze 1856, 1863; Eckhard 1858; Babuchin 1872; Paschutin 1873; Brunn 1875). Comprehensive work on different species by Max Schultze firmly established that the cellular organization of the amphibian olfactory system (OS) showed a high similarity to other vertebrates (Schultze 1856, 1863; reviewed by Zippel 1993). Thus, frogs were readily recognized as important models to understand general cellular organization and function of the vertebrate OS. Application of electron microscopy allowed to extend the morphological information about the cell types of the OS with ultrastructural details. This technical progress, for instance, revealed that the apical appendages of receptor neurons, i.e., cilia, are the cellular subcompartment of odor molecule binding and transduction (Bloom 1954). In particular, the physiology of receptor neurons in the OE was heavily investigated. Slow odor-evoked electrical signals were recorded in receptor neurons and the olfactory bulb (OB) was established as an important relay station for olfactory information (Ottoson 1956, 1971; Gesteland et al. 1965; Kauer 1974). Many underlying principles of vertebrate olfactory transduction and odor processing in general were derived from pioneering work in anurans (Takagi and Shibuya 1960; Shibuya et al. 1962; Lancet 1986; Duchamp-Viret and Duchamp 1997; Schild and Restrepo 1998).

**Fig. 1:**
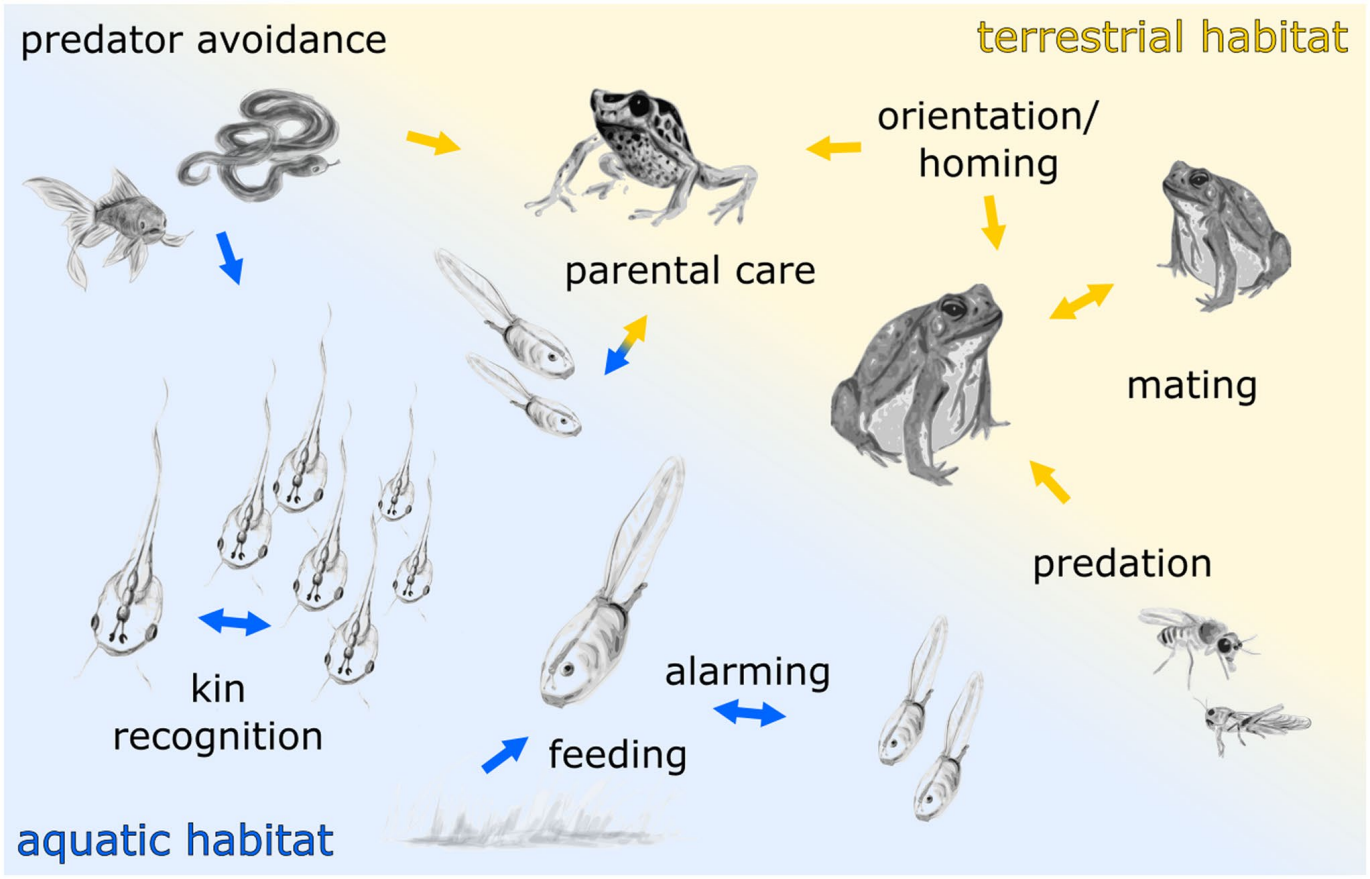
Overview of anuran behaviors involving the sense of smell. Anurans use waterborne (blue arrows) and airborne (yellow arrows) odor molecules for a multitude of behaviors during different life-stages. Based on the diversity of anuran habitats, odor-guided behaviors vary extensively between species

Despite the wealth of information gathered about OS structure and function, only little was known about the behavioral relevance of olfaction in anuran amphibians. Early attempts to study the role of olfaction in frogs and toads were unsuccessful and did not obviously support a prominent role in their lifestyle, e.g., in foraging (Risser 1914). Because of these early hindrances, olfactory research was notably delayed in anuran amphibians and not until much later, behaviors were identified that particularly depend on the sense of smell (Fig. 1). Instead, many researchers shifted their focus towards olfaction in urodele amphibians (salamanders), where the olfactory sense more obviously played a major role in their lifestyle. Intraspecific interactions via chemical communication emerged as a promising research field and have been very well studied over time (Woodley 2010, 2014, 2015). Up to now, chemical communication in anurans has been investigated less but may play a more important role than appreciated so far (Belanger and Corkum 2009; Waldman 2016; Woodley 2010, 2014, 2015).

## “Water-to-land” transition in evolution, development, and individual lifestyle

Anurans occupy a unique position in the vertebrate lineage as a group that is heavily exposed to the interface between terrestrial and aquatic environments (Reiss and Eisthen 2008). This applies on multiple axes: (i) phylogenetically as a group sharing traits with the first tetrapods that conquered the terrestrial environment in evolution, (ii) ontogenetically with a development that includes aquatic and terrestrial stages connected via metamorphic remodeling, and (iii) individually with common changes in habitat during the individual lifecycle. In the following, we describe the specific challenges for the sense of smell, and thus the OS, along these three axes.

### Evolution: olfaction in water vs. olfaction in air

The evolutionary shift in the vertebrate lineage from an aquatic to a terrestrial environment is challenging in many physiological aspects and necessitated substantial adaptations, including modified respiratory mechanisms and sensory systems (Schoch 2014; Janes et al. 2019). In amphibians, exposure of the olfactory surface to odor molecules is tightly coupled to oscillatory movements of the floor of the mouth (Bruner 1914; reviewed by Jørgensen 2000). Thereby, two modes of respiratory movements can be identified, infrequent strong contractions to pump air into the lungs, and shallow contractions to circulate air or water into the nasal cavity to allow olfactory detection (Gargaglioni and Milsom 2007).

Depending on the medium that the OS is exposed to, different physical and chemical constraints apply before odor molecules can successfully interact with olfactory receptors on receptor neurons. Important factors that differ in the two environments are olfactory medium density, viscosity, diffusion speed, and solubility of odor molecules (Hemilä and Reuter 2008). Higher density and viscosity of water, coupled with a much lower diffusion speed in comparison to air, make transport of odor molecules to the sensory surface a formidable challenge in aquatic environments (Tierney 2015). Aquatic organisms have evolved mechanisms to facilitate water displacement onto the olfactory sensory surface, including nostril structure, streamlined internal cavities, and actively beating cilia in the nasal cavities (Föske 1934; Cox 2008; Reiten et al. 2017).

The range of molecules mainly carried by water varies considerably from molecules carried by air and thus aquatic and terrestrial animals are generally exposed to very different classes of odorants (Hemilä and Reuter 2008). Water easily dissolves hydrophilic molecules, ranging from small organic molecules to large proteins, and thus makes them accessible to aquatic olfactory organs. In air, on the other hand, the volatility of odor molecules plays a decisive role in the ease of dispersion in the medium (Eisthen and Schwenk 2008). Highly volatile molecules are distributed easily in air and are thus preferentially accessible to the olfactory organs of terrestrial animals. During tetrapod evolution, the OS segregated into subsystems and amphibians feature an accessory OS in addition to the main OS (Taniguchi and Taniguchi 2014). The accessory OS is made of the sensory epithelium of the vomeronasal organ (VNO), the accessory OB, and higher brain centers (Mohrhardt et al. 2018). The VNO is fluid-filled, has no direct contact to the aerial environment and is thought to be specialized for the detection of odor molecules with lower volatility (Reiss and Eisthen 2008). Alternatively, molecules that cannot be freely distributed in the medium (i.e., hydrophobic molecules in aqueous medium and non-volatile molecules in air) can be sensed through direct physical contact with the olfactory organ or in particular the vomeronasal organ, e.g., by licking or touching (Hemilä and Reuter 2008).

**Fig. 2:**
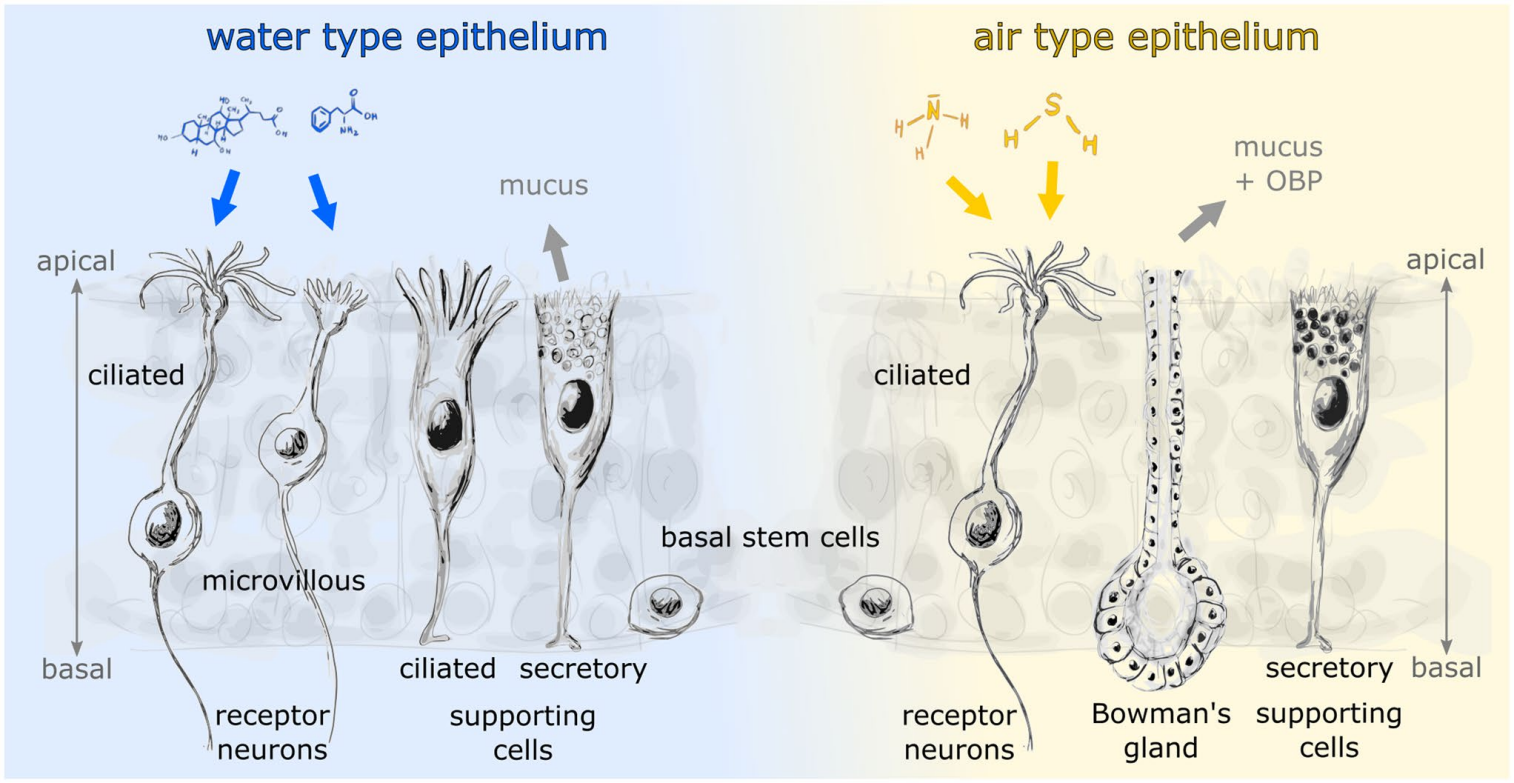
Structure of the anuran main olfactory epithelia and the adaptations to aquatic and terrestrial environments. The cellular components of a water-type (left) and air-type (right) olfactory epithelium are shown. While the water epithelium contains both ciliated and microvillous receptor neurons, only ciliated receptor neurons are found in the air type epithelium. The water epithelium has two types of supporting cells (ciliated and secretory), compared to only secretory supporting cells in the air epithelium. Mucus in the water- and air-epithelium is mainly produced by secretory supporting cells and Bowman’s glands, respectively. Both epithelial types include a population of basal stem cells near the basal lamina. OBP olfactory binding protein

Olfactory epithelia of both air and water specialized olfactory organs are lined with a thin mucus layer (Getchell and Getchell 1992). Consequently, odor molecules must overcome the mucus barrier to reach the olfactory receptors on receptor neurons. Certain mucus properties seem to be beneficial or even essential for air olfaction, as specialized mucus-secreting Bowman’s glands are found solely in air-exposed OE (Fig. 2). In fishes and larval amphibians, the mucus is produced by various cell types of the OE (Getchell and Getchell 1992; Menco and Farbman 1992). In terrestrial vertebrates (and interestingly also in insects), the mucus contains so-called odorant binding proteins that facilitate mucus solubility of odor molecules and may also serve other functions as well (Pelosi 1994; Sun et al. 2018).

In conclusion, it seems plausible that air-exposed noses presented an evolutionary opportunity for early terrestrial organisms to specialize for the detection of volatile odor molecules. This seems to have led to the development of specialized olfactory surfaces that are preferentially tuned to odor molecules with certain chemical properties, such as volatility and solubility. These properties are more relevant than the carrier medium itself (Hemilä and Reuter 2008). Adaptation of the OS to these two environments is particularly evident in the OS of different stages of the anuran life cycle.

### Ontogenetic development

A unique characteristic shared by most anuran amphibians is their biphasic life cycle featuring morphologically distinct tadpole and frog stages that are highly adapted for vastly different lifestyles, mostly connected to aquatic and terrestrial habitats (McDiarmid and Altig 1999; Handrigan and Wassersug 2007; Elinson and Pino 2012). The underlying body plan of anuran larval stages is quite dissimilar to adult stages and serves as foundation for a large ecological diversity of tadpole subtypes (Altig and Johnston 1989; Roelants et al. 2011). The OS of tadpoles and frogs specifically evolved to suit the prevailing aquatic or terrestrial environment, respectively.

A shared characteristic of free living tadpoles is a body plan with specialized feeding apparatus to exploit an aquatic habitat with rich primary food sources (Wassersug 1989). The aquatic habitat is however not uniform and tadpoles have radiated into several microhabitats (McDiarmid and Altig 1999; Roelants et al. 2011). In an attempt to describe morphology as a function of habitat, Altig and Johnston (1989) have sub-categorized several ecomorphological guilds of tadpoles. They primarily describe feeding related types e.g., the benthic type, which relies on rasping off vegetation from the ground, carnivorous tadpoles or filter feeders, but also categorized classes of tadpoles living in still water (lentic) vs. streams and rivers (lotic) (Altig and Johnston 1989). The temporary nature of most tadpole habitats in terms of food/water supply and predatory risk also favors a rapid transition to the adult stage via metamorphosis.

Metamorphosis is a shared evolutionary strategy for amphibians reflecting a broad range of larval adaptations to environmental parameters in parallel to and largely independent adaptations of adult stages (Fritzsch 1990; Reiss 2002). During metamorphosis, many tissues undergo drastic changes and the OS is remodeled to satisfy the demands of terrestrial olfaction (Dodd and Dodd 1976; Hansen et al. 1998; Gascuel and Amano 2013; Jungblut et al. 2017). This includes general modifications of OE properties, like the emergence of mucus-producing Bowman’s glands (Taniguchi et al. 1996; Hansen et al. 1998; Jermakowicz et al. 2004; Wang et al. 2008). In line with this, no odorant binding proteins have been found in tadpoles (just like in fish), but expression only begins in the developing air nose during metamorphosis (Millery et al. 2005). Other changes are a shift in receptor neuron subtypes with different morphologies, transduction machinery, olfactory receptor protein expression, and odor sensitivities (Taniguchi et al. 1996; Hansen et al. 1998; Jungblut et al. 2011; Dittrich et al. 2016; Syed et al. 2017). Thus, the pre- and postmetamorphic OS each are very likely tuned to fulfil distinct important tasks in an ecological context.

### Individual lifestyle

Anurans are a highly diverse group that has conquered many ecological niches and the OS contributes to detection of food sources and predators, orientation, alarm signaling, and intraspecific interactions (Fig. 1). Interestingly, tadpole and adult stages have undergone separate evolutionary modifications to their individual environmental parameters (Fritzsch 1990; Reiss 2002). The distinct habitats of larval and adult anurans demand different tunings and adaptations of their sensory systems (Duellman and Trueb 1994; Wells 2007a). While the adult frog has to establish a complex long-term relationship with its environment to successfully reproduce, the anuran tadpole is a temporary inhabitant of freshwater ecosystems with the sole goal to make it to metamorphosis unscathed (McDiarmid and Altig 1999; Wells 2007a). This dictates the major challenges a tadpole has to face: feeding to optimize growth while evading the risk of predation, both of which heavily involve the animal’s sense of smell (Hoff et al. 1999).

#### Role of olfaction in larval anurans

While tadpoles were traditionally viewed as being herbivorous or feeding on degraded organic materials, this dogma is presently challenged and tadpoles are mostly regarded as opportunistic omnivores (Hoff et al. 1999; Altig et al. 2007). Tadpoles of species breeding in small and often ephemeral ponds in bromeliads with a low availability of food rely on trophic eggs provided by their mothers (Weygoldt 1980; Schulte 2014) or resort to cannibalism in the event of food shortage (Crump 1983; Poelman and Dicke 2007; Masche et al. 2010). Additionally, some ranid tadpoles, e.g., show that members of the same species can be facultatively carnivorous (Schiesari 2004) or herbivorous (Pryor and Bjorndal 2005), suggesting spatiotemporal changes in feeding ecology. Data on foraging behavior in anuran tadpoles is mostly inferred from the morphology of their mouthparts and buccopharyngeal space (Orton 1953; Wassersug 1980; Altig and Johnston 1989; McDiarmid and Altig 1999). The presence of keratinized mouthparts enables some tadpoles to rasp off nutritional particles from the vegetation or other animals, which are then filtered by special branchial food traps (Orton 1953; Wassersug 1972; Altig and Johnston 1989). In obligate filter feeding tadpoles, such as most pipid species, the buccopharyngeal space is enlarged to optimize food entrapment (Wassersug 1972; Seale 1982). While these mechanisms are well described, almost no reports on the sensory correlates of tadpole foraging are known.

Tadpoles are generally considered severely nearsighted (Mathis et al. 1988; Hoff et al. 1999) and often inhabit murky, heavily vegetated, shaded, or turbulent water with low visibility (McDiarmid and Altig 1999; Wells 2007a), so foraging based solely on visual cues is quite improbable. In one of the only experimental studies on the matter, it was shown that *Rana temporaria* tadpoles prioritize chemical (olfactory) cues over visual cues to detect food sources (Veeranagoudar et al. 2004). In contrast to the lack of chemical food cues, the absence of visual food cues did not alter foraging behavior (Veeranagoudar et al. 2004). Considering the high variability of freshwater habitats and the associated trophic diversity, it seems likely that a lot remains to be discovered about olfaction-mediated foraging behavior (Altig and Johnston 1989; Alford 1999).

During the search for food, tadpoles are making themselves susceptible to predation. Conversely, extended periods of hiding without feeding will delay growth and eventually metamorphosis (Lima and Dill 1990). Several levels of predation risk assessment are thus in place and mostly chemical in nature (Chivers and Smith 1998; Ferrari et al. 2010; Hettyey et al. 2015). Upon detection of a predator-odor, anuran tadpoles generally decrease activity and display increased refuge-seeking behavior (Petranka et al. 1987; Hews 1988; Kats et al. 1988; Stauffer and Semlitsch 1993; Manteifel 1995; Griffiths et al. 1998; Hoff et al. 1999; Marquis et al. 2004). This behavior has been shown to vary according to the species and the predator. For instance, tadpoles of the midwife toad *Alytes muletensis* showed predator-avoidance in response to odors emitted by a sympatric snake species, but not to an allopatric species (Griffiths et al. 1998). Similarly, tadpoles of several species behaviorally responded to the presence of their native larval dragonfly predator, but not to the presence of a novel crayfish predator (Nunes et al. 2013). The same study also shows that not only the familiarity of a predator but also its diet has an effect on tadpole behavior. Tadpoles responded with an avoidance behavior also to the novel exotic predator, if it had already successfully fed conspecific tadpoles (Nunes et al. 2013). Such postdigestive chemical alarm cues have been described in multiple sources and it is predicted, that stronger antipredator behaviors are elicited when closely related species or conspecifics are preyed on (Wilson and Lefcort 1993; Chivers and Mirza 2001; Marquis et al. 2004; Schoeppner and Relyea 2005; but see Scribano et al. 2020).

Alarm cues of conspecifics are not necessarily released after being eaten by a predator but have also been shown to be emitted from the skin via damage in bufonid tadpoles (Eibl-Eibesfeldt 1949; Kulzer 1954; Pfeiffer 1966). The alarm cues (Schreckstoff) were first described in fishes (v. Frisch 1942) and have been shown to be present in most bufonid species (Kulzer 1954; Hews 1988), while being absent, e.g., in pipids (Pfeiffer 1966). Mechanical crushing of conspecifics also elicited fright reactions in cane toad *Chaunus (Bufo) marinus* tadpoles (Hagman and Shine 2008) and the red-legged frog *Rana aurora* (Wilson and Lefcort 1993). In ranid tadpoles, it has also been shown that peptide-cues can even be actively secreted by skin cells upon predator attack as an alarm substance for conspecifics (Fraker et al. 2009). In addition to these attack- or capture-related cues, *Rana aurora* tadpoles have been shown to release disturbance-cues upon detection of predators in their vicinity (Kiesecker et al. 1999). The major component of this disturbance-signal is probably a high concentration of ammonia-excretion via the urine (Kiesecker et al. 1999), which seems to function as a general alertness-signal not only to conspecifics but also across prey species in the same ecosystem (Manteifel and Kiseleva 2011). A persistent exposure to predator alarm cues has been shown to even induce morphological changes (Buskirk and Relyea 1998; Schoeppner and Relyea 2005) or change tadpole life history and lead to, e.g., premature hatching (Chivers and Mirza 2001).

Both foraging as well as predator-avoidance in tadpoles are supported and facilitated by various forms of social behavior and most of these behaviors also probably rely on the sense of smell (McDiarmid 1978; Roche 1993; Hoff et al. 1999; Roland and O’Connell 2015). The most obvious social behavior observable in many tadpole species is schooling (Waldman 1991; Hoff et al. 1999). Tadpole aggregations can be situational/asocial (e.g., due to a common food source) or social due to identifying and associating with conspecifics (Wassersug 1973; Waldman 1991; Hoff et al. 1999). Tadpoles that generally form larger groups like the Cascade frogs *Rana cascadae* (Blaustein and O’Hara 1982) or the American toad *Bufo americanus* (Waldman and Adler 1979) were shown to identify kin and preferentially associate with siblings over non-siblings (Waldman and Adler 1979; Blaustein and O’Hara 1982). Conversely, species that do not generally form schools failed to display kin recognition (Fishwild et al. 1990). Kin recognition did not rely on vision or sound and putatively is conveyed via waterborne odor cues (Blaustein and O’Hara 1982; Waldman 1985; Eluvathingal et al. 2009). A nasal occlusion of *Bufo americanus* tadpoles completely eradicated kin-recognition, which suggests that olfaction is required for this social behavior (Waldman 1985). A possible genetic basis for kin recognition in tadpoles of the African Clawed frog *Xenopus laevis* is the self-referent recognition of peptides of the major histocompatibility complex (MHC 1) (Villinger and Waldman 2008). Similarly, in rodents MHC peptides are involved in mate recognition (Leinders-Zufall 2004).

The recognition of kin can have various advantages for the individual tadpoles (Roche 1993; Hoff et al. 1999). Grouping together signifies that the time and resources spent in vigilance to detect predators is shared (O’Hara and Blaustein 1981), which means the individual can more efficiently concentrate on feeding and have a better chance to survive predation risks (Roche 1993). In times of food scarcity, groups of siblings produce differently sized individuals to ensure survival of at least some tadpoles, while all tadpoles are smaller in non-sibling groups (Blaustein and Waldman 1992). Concerning cannibalistic tadpoles, kin recognition facilitates predation of non-related tadpoles over kin (Roche 1993). In the polymorphic spadefoot toad *Scaphiopus bombifrons*, which often displays a herbivorous and a carnivorous tadpole morphotype, carnivorous tadpoles preferentially preyed on non-siblings unless they were very hungry (Pfennig et al. 1993).

In addition to the chemical basis of kin recognition, there is some evidence that there is also chemical communication between some tadpoles and their parents (Kam and Yang 2002). Breeding in small temporary ponds comes with the advantage of less predation but also lower food abundance (Wassersug et al. 1981). This favored the evolution of parenting behavior and food provisioning in many tropical frog species (Wells 2007a; Brown et al. 2008; Roland and O’Connell 2015; Schulte et al. 2020). In the Taiwanese tree frog *Chirixalus eiffingeri*, tadpoles become more active when exposed to water conditioned by a female frog (Kam and Yang 2002), possibly in the anticipation of trophic eggs laid by the female. The visual presence of a female frog did not elicit the same response, suggesting chemical communication as the trigger for the behavior of tadpoles (Kam and Yang 2002).

#### Role of olfaction in adult anurans

After a tadpole successfully survives until metamorphosis and transforms into a juvenile frog, a new and complex sensory interaction with the environment starts (Wells 2007a). The historical notion that anuran behaviors like foraging or mating choice are unimodally relying on vision or vocalization/audition has been replaced by a more integrative approach (Starnberger et al. 2014a). Complex behavior results from information perceived through multiple sensory channels, including olfaction, that can either be complementary or redundant (Ferguson 1971; Sinsch 2006; Starnberger et al. 2014b). While visual motion detection is still considered the major player in anuran foraging behavior, other senses like olfaction or tactile senses can modulate (Michaels et al. 2018) or functionally replace visual input (Kramer 1933; Altner 1962; Shinn and Dole 1978, 1979a).

Olfactory-guided prey approach and feeding-related behaviors like tongue protrusions have been demonstrated in terrestrial species like the leopard frog *Rana utricularia* (Shinn and Dole 1978) and some bufonid species (Heusser 1958; Shinn and Dole 1979a, 1979b). In pipid frogs, which inhabit murky waters, olfaction has been suggested to play a role in detecting prey when it is still far away (Kramer 1933; Altner 1962), while water perturbations and vibrations caused by prey animals are the proximate stimuli inducing foraging behavior (Kramer 1933; Elepfandt et al. 2016). While it is vital to find food sources, it is equally important for anurans to evade predation themselves.

Vision seems to be the most important sense to detect predators (Kramer 1933; Heinen 1994; Wells 2007b); however, several instances of olfactory-mediated predator avoidance behaviors are documented (Flowers and Graves 1997; Gonzalo et al. 2006; Hamer et al. 2011). Juvenile bufonids were shown to detect and avoid odors from garter snakes, their natural predator (Flowers and Graves 1997), and the presence of snake odors were shown to influence pond choice in juveniles of the Iberian green frog *Rana perezi* (Gonzalo et al. 2006). Adult great barred frogs *Mixophes fasciolatus* were generally shown to be attracted to odors of conspecifics to form aggregations (Hamer et al. 2011). When snake odors were mixed to the odors of conspecifics, their attraction was decreased, suggesting that they would rather aggregate with conspecifics if there was no risk of predation (Hamer et al. 2011). Eat, while not eaten is a credo relevant for all frogs. Adult anurans however have occupied a range of diverse habitats and behaviors which involve different sensory modalities to a different degree and for different behavioral tasks (Wells 2007a).

Even though most anurans forage and move on land, their reproductive success heavily depends on the presence of water. Anuran species living in temperate climatic regions like the common toad *Bufo bufo* mostly rely on permanent bodies of water for breeding and usually return to their home pond, even when other ponds are in their proximity (Heusser 1960). Orientation behavior in anurans involves many different senses working in an at least partially redundant manner (for review, see Ferguson 1971; Sinsch 2006). Field studies showed that toads navigated to their home pond after spatial displacement even when visual and magnetic senses were experimentally impaired (Jungfer 1943; Sinsch 1987). Anosmic frogs however showed difficulty in locating their home ponds (Sinsch 1987; Ishii et al. 1995). Choice assay studies further supported the idea that natal pond odors are preferred over odor cues from other ponds (Grubb 1973a, 1973b, 1975, 1976; Forester and Wisnieski 1991), although olfactory-guided orientation was not demonstrated for all examined species (Grubb 1973b). As shown for other animals like salmon or turtles, a growing body of evidence suggests, that anurans also learn to recognize pond odor via imprinting during larval stages (Ishii et al. 1995; Ogurtsov and Bastakov 2001; Ogurtsov 2007). Tadpoles raised in water containing an artificial chemical marker were able to recognize the marker in a binary choice assay after metamorphosis, while non-imprinted juveniles were indifferent to the cue (Ogurtsov 2007). Whether the learned home-odor is perceived as attractant also depends on the life stage of the frogs or their seasonal change in behavior (Sinsch 1988; Reshetnikov 1996; Shakhparonov and Ogurtsov 2003). After the start of dispersal phase from their home pond, juvenile wood frogs *Lithobates sylvaticus* (Popescu et al. 2012) and the green toad *Bufo viridis* (Shakhparonov and Ogurtsov 2003) temporarily showed no preference for the odor of their home pond.

While most frogs living in temperate regions usually deposit their eggs in big clutches and barely provide parental care, many tropical frogs breed in ephemeral water bodies in bromeliads and guard their eggs or shuttle their tadpoles to new pools if food resources are scarce (Brown et al. 2008; Roland and O’Connell 2015). To avoid food competition and predation in these small water bodies, poison dart frogs belonging to the genus *Ranitomeya* were shown to inspect the oviposition site before laying their eggs (Weygoldt 1980; Brown et al. 2008; Schulte et al. 2011). In a choice experiment, parental *Ranitomeya variabilis* were demonstrated to recognize the presence or absence of other anuran larvae in small water pools using their chemical senses (Schulte et al. 2011). Since already present tadpoles could potentially feed on the eggs, they avoided deposition in pools containing chemicals from other tadpoles. Furthermore, they did not shuttle larval tadpoles to pools containing chemical cues from cannibalistic tadpoles, while they did not avoid pools containing cues from non-cannibalistic tadpoles (Schulte et al. 2011). Other than the examination of potential oviposition sites, odors were also hypothesized to play a role in detecting novel bromeliad pools for the brilliant-thighed poison frog *Allobates femoralis* (Pašukonis et al. 2016).

Homing and navigating behaviors in anurans are often closely related to finding a mate and reproduction. While it has been shown that chemical communication among salamanders is probably the major sensory channel to facilitate reproduction (Houck 2009; Woodley 2015), vocal communication was long considered the only mode of communication and mate-finding in anurans (Starnberger et al. 2014a). However, in the course of the last 20 years, the involvement of both waterborne (Wabnitz et al. 1999; Pearl et al. 2000) and airborne chemicals (Poth et al. 2012; Starnberger et al. 2013) in frog communication has been demonstrated. A peptide produced by the parotoid and rostral glands of the males of the magnificent tree frog *Litoria splendida* called splendipherin attracts females of the same species and was the first identified frog sex pheromone (Wabnitz et al. 1999). Similarly, male dwarf African clawed frogs (genus *Hymenochirus*) use secretions of their skin glands (breeding glands) to attract females (Pearl et al. 2000). In the Australian terrestrial toadlet *Pseudophryne bibronii*, odors from both sexes can act as attractants (Byrne and Keogh 2007). Female odors were furthermore shown to increase calling behavior in male frogs, thus this species probably uses a bi-modal communication system (Byrne and Keogh 2007). A tri-modal system involved in mate recognition and courtship is suggested for the African reed frogs (Hyperoliidae) (Starnberger et al. 2014b). Males of this family have gular glands on their often colorful vocal sacs, which produce sex and species-specific chemical cocktails (Starnberger et al. 2013; Menke et al. 2016, 2018; Melnik et al. 2019). The exact interplay between visual, auditory, and chemical cues in mating behavior are however not fully understood. A similar cocktail of chemicals was obtained from the femoral glands of male mantellid frogs endemic to Madagascar (Poth et al. 2012; Menke et al. 2016). Some volatile compounds also showed effects on the behavior, presumably linked to mating (Poth et al. 2012). Apart from mostly sex-attractant pheromone systems, odorous peptides from the mountain chicken frog *Leptodactylus fallax* (King et al. 2005) and the Australian toadlet *Pseudophryne bibronii* (Byrne and Keogh 2007) were demonstrated to initiate fighting behavior between males of the respective species, possibly during the onset of mating behavior.

While in some species, pheromone-mediated communication might be complementary to vocalizations, species living in noisy environments might rely more heavily on olfaction to find reproductive partners (Asay et al. 2005). The coastal tailed frog *Ascaphus truei* lacks a tympanic membrane and vocal cords in addition to being nocturnal and living near noisy fast-moving streams (Noble and Putnam 1931; Metter 1967; Asay et al. 2005). In consequence, chemical cues seem to be the major mediator to find and recognize mating partners (Asay et al. 2005). Since habitat and reproductive modes in anurans vary extensively (Wells 2007a), the occurrence of thus far unknown, species-specific ways of chemical communication are awaiting their discovery.

## The anuran olfactory system

Based on the ecological and behavioral data presented above, both waterborne and airborne odorants play a vital role in the life of anuran amphibians. However, there is a huge knowledge gap between the ecology and the neuronal circuits involved. The peripheral organ is the by far best studied part of the anuran OS and detailed information about the anatomy, the morphological neuron types and olfactory receptor expression is available. For a comprehensive review and a comparative inspection of the olfactory periphery of different anuran species, we refer the reader to Jungblut et al. (2020) that is included in the same special issue as this article. The following part of this review aims at describing the general properties of the anuran OS on multiple levels and putting them into an evolutionary context. We will particularly focus on cellular, molecular, and circuitry features that are potentially important adaptations for olfaction in air vs. water environments.

### Segregation into functional subsystems

The general organization of the OS in all groups of the vertebrate lineage is astonishingly similar. They are all composed of (i) a peripheral OE, which is part of the olfactory mucosa and functions as detection site of odor molecules, (ii) an OB serving as a first relay center of the system, and (iii) several terminal processing centers, i.e., olfactory cortices (Ache and Young 2005; Eisthen and Polese 2007; Illig and Wilson 2009; Taniguchi et al. 2011). The anatomy, cellular composition, and functioning of the first two stages of the OS differ only slightly between species (Graziadei 1971; Taniguchi and Taniguchi 2014; Bear et al. 2016). The third stage, i.e., the cortical area, on the other hand, is more variable between vertebrate groups. One reason for this may be that the homology of cortical areas of non-mammalian vertebrate lineages and the ones identified in mammals is still unclear (Illig and Wilson 2009).

**Fig. 3:**
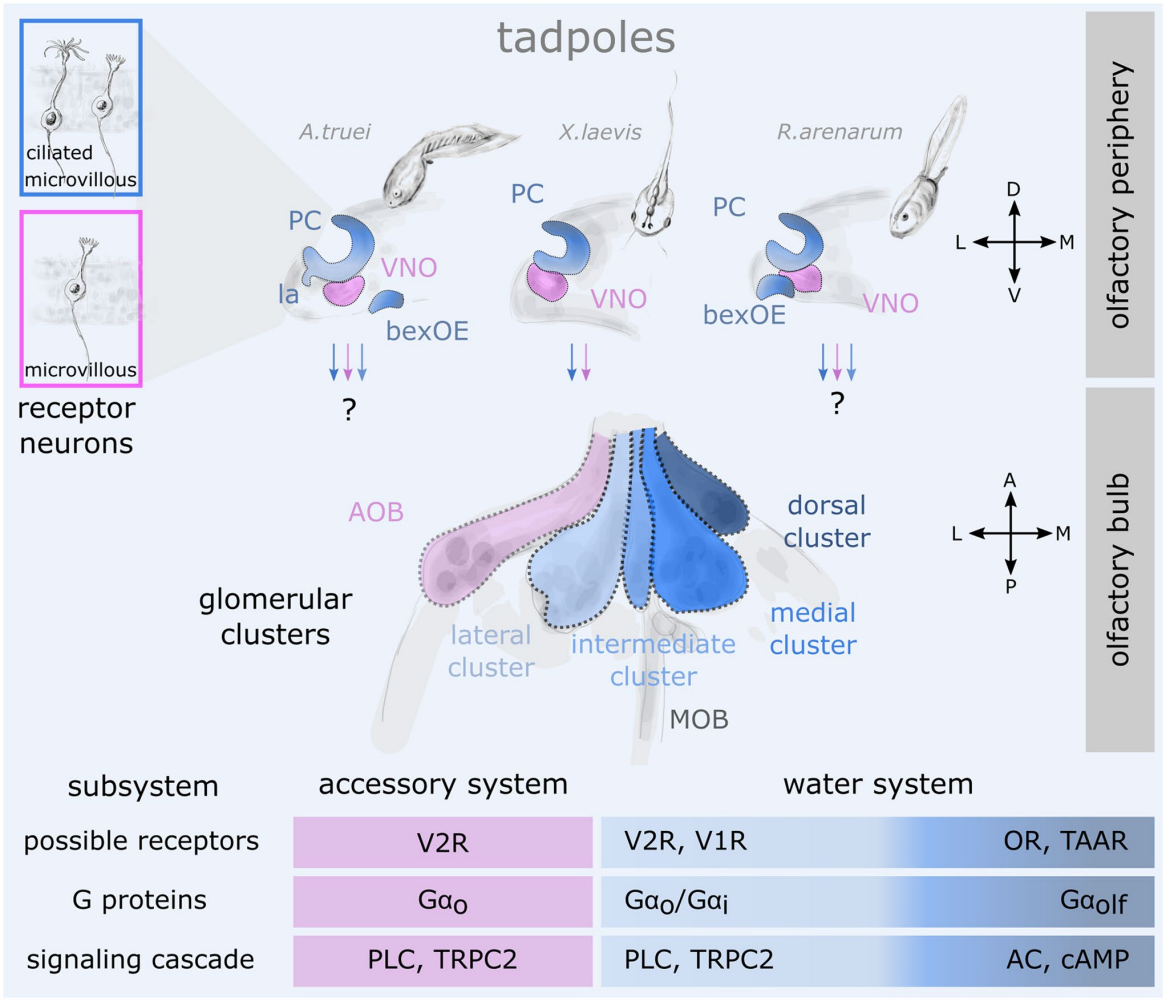
Anatomical, cellular, and molecular characteristics of the olfactory system in larval anurans. Above: Schematic coronal section of the left olfactory organs in the nose are shown for an archaeobatrachian (*Ascaphus truei*), a mesobatrachian (*Xenopus laevis*) and a neobatrachian tadpole (*Rhinella arenarum*). The main olfactory system (blue) consisting of the principal cavity epithelium (PC), the lateral appendix (la), and the buccal exposed epithelium (bexOE) differs between the species, while all three have an anatomically separate vomeronasal organ (VNO; purple). The boxes on the left show the types of receptor neurons present in each organ. Middle: The respective axonal projections of the receptor neurons and their glomerular targets in the olfactory bulb show an anatomical segregation into glomerular clusters. Only the left hemisphere is shown. While the VNO projects to the accessory olfactory bulb (AOB, purple), the main olfactory epithelium projects to four clusters in the ventral main olfactory bulb (MOB; shades of blue). The respective projection targets of the bexOE and the la within the glomerular array are still unclear. Below: The putative molecular components linked to the above described olfactory subsystems are summarized based on data from the genus *Xenopus* and might show some inter-species variation that still needs to be uncovered. A anterior, AC adenylate cyclase, AOB accessory olfactory bulb, bexOE buccal exposed olfactory epithelium, cAMP 3′,5′-cyclic adenosine monophosphate, D dorsal, L lateral, la lateral appendix, M medial, MOB main olfactory bulb, OR OR type olfactory receptor, P posterior, PC principal cavity, PLC phospholipase C, TAAR trace amine-associated receptor, TRPC2 transient receptor potential channel 2, V ventral, V1R type 1 vomeronasal receptor, V2R type 2 vomeronasal receptor, VNO vomeronasal organ

**Fig. 4:**
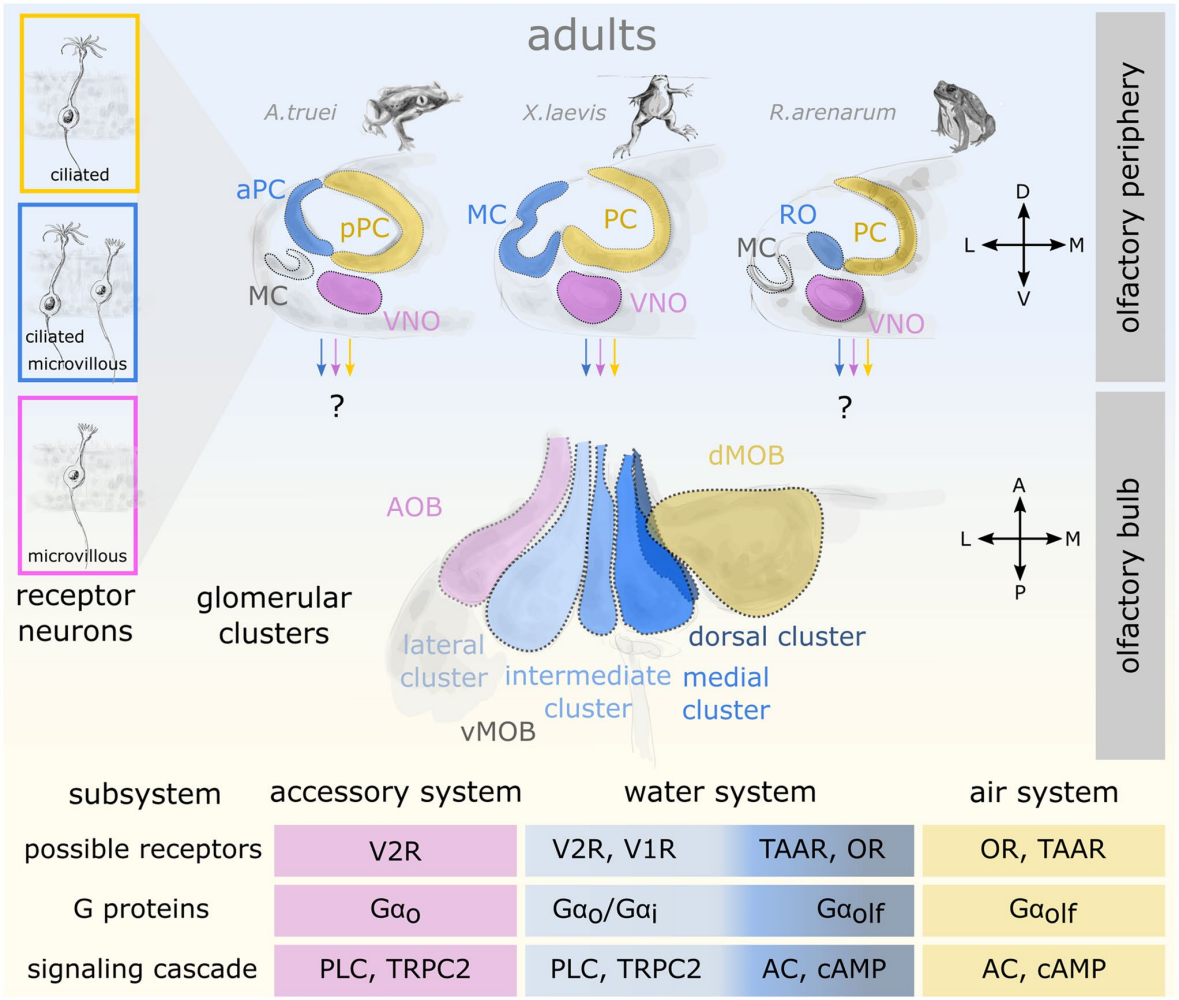
Anatomical, cellular, and molecular characteristics of the olfactory system in adult anurans. Above: Schematic coronal section of the left olfactory organs in the nasal cavities are shown for an archaeobatrachian (*Ascaphus truei*), a mesobatrachian (*Xenopus laevis*), and a neobatrachian frog (*Rhinella arenarum*). The main olfactory epithelium in adult anurans is segregated into a water- (blue) and an air-portion (yellow). While a major part of the principal cavity (PC) is lined with an air-type epithelium, the water-type epithelium is situated in different anatomical structures in the depicted species: anterior portion of the PC (aPC), middle cavity (MC), and recessus olfactorius (RO). The MC is non-sensory in most anurans. All species possess a vomeronasal epithelium in the VNO (purple). The boxes on the left show the types of receptor neurons present in each of the three epithelia. Middle: The respective axonal projections of the receptor neurons and their glomerular targets in the olfactory bulb show an anatomical segregation. Only the left hemisphere is shown. Due to incomplete comparative data, the scheme depicts the connectivity only for the genus *Xenopus*. While the VNO projects to the accessory olfactory bulb (AOB), the water-portion of the main olfactory epithelium (MC) projects to at least four clusters on the ventral surface of the main olfactory bulb (vMOB; shades of blue). The air-portion connects to the dorsal MOB (dMOB). Below: The putative molecular components linked to the above described olfactory subsystems are summarized based on data from the genus *Xenopus* and might show some inter-species variation that still needs to be uncovered. A anterior, AC adenylate cyclase, AOB accessory olfactory bulb, aPC anterior principal cavity, cAMP 3′,5′-cyclic adenosine monophosphate, D dorsal, dMOB dorsal main olfactory bulb, L lateral, la lateral appendix, M medial, MC middle cavity, MOB main olfactory bulb, OR OR type olfactory receptor, P posterior, PC principal cavity, PLC phospholipase C, pPC posterior principal cavity, RO recessus olfactorius, TAAR trace amine-associated receptor, TRPC2 transient receptor potential channel 2, V ventral, V1R type 1 vomeronasal receptor, V2R type 2 vomeronasal receptor, vMOB ventral main olfactory bulb, VNO vomeronasal organ

In contrast to the singular OS present in fishes, consisting of a single OE and an undivided OB, the OS in most tetrapods is generally defined by anatomically segregated olfactory subsystems (Figs. 3 and 4). Chief among them, the main OS consists of the main OE and the main OB and the accessory OS is made of the epithelium of the VNO and the accessory OB (Trotier and Doving 1998; Munger et al. 2009; Bear et al. 2016; Fleischer et al. 2020). These two subsystems feature distinct molecular and functional characteristics and are considered ancestral traits of the tetrapod lineage (Taniguchi et al. 2011; Taniguchi and Taniguchi 2014). The initial idea of the VNO as an exclusively pheromone-detecting apparatus in addition to the odorant-detecting main OE proved oversimplified in the light of modern neurobiology (Eisthen 1992, 1997). It is now clear that social olfactory cues can be also detected by the main OE and that the main and accessory OS can even serve parallel, partly overlapping functions (Mohrhardt et al. 2018). A promising hypothesis is that the two systems are instead tuned for distinct physicochemical properties of odor molecules, like volatility and solubility (Holy 2018).

Since an anatomically distinct VNO was only found in terrestrial tetrapods, it was first considered an adaptation to life on land (Bertmar 1981). This theory proved inconclusive though, because the VNO is also present in the aquatic larvae of amphibians (Fig. 3) and a primordial VNO is already present in earlier diverging vertebrates, like lungfish (Eisthen 1992; González et al. 2010; Nakamuta et al. 2012; Chang et al. 2013). In all three extant amphibian lineages, the VNO seems to be an ancestral trait in larval as well as adult animals (Schmidt and Wake 1990; Eisthen 1992; Jermakowicz et al. 2004; Benzekri and Reiss 2012; Jungblut et al. 2012). A notable exception is the proteid family of urodele amphibians that have secondarily lost the VNO (Eisthen 2000). In caecilians, the VNO is connected to tentacles on the head of the animals and is supposedly used during burrowing and diving, while the main nasal cavity remains closed (Schmidt and Wake 1990). It is hypothesized that the caecilian VNO at least partially assumes the role of a water nose, while the main OS seems mainly involved in volatile odorant detection (Schmidt and Wake 1990).

In urodele and anuran amphibians, the main OS is thought to detect both waterborne and airborne odorants (Reiss and Eisthen 2008). The main OE of urodeles consists of a single cavity that differs morphologically in aquatic and terrestrial species or between aquatic and terrestrial life-stages within individuals (Różański and Żuwała 2019). The peripheral olfactory organ of adult anurans is generally more complex, exhibiting an interconnected chamber system that has been described in detail for many species (Fig. 4; Föske 1934; Helling 1938; Paterson and Hindle 1951; Jermakowicz et al. 2004; Jungblut et al. 2011, 2017; Benzekri and Reiss 2012; Nowack and Vences 2016; Quinzio and Reiss 2018). A large medial diverticulum is known as the principal cavity (PC) (Föske 1934; Bloom 1954; Reese 1965; Menco 1980; Mair et al. 1982; Reiss and Eisthen 2008) and serves as an air nose. The presence of an elevated ridge on the floor of the PC (eminentia olfactoria) was hypothesized to be a feature correlated with a more terrestrial lifestyle in some anurans (Helling 1938; Quinzio and Reiss 2018).

Most anurans additionally exhibit a segregated portion of the main OE dedicated to aquatic olfaction, the adult water nose (Fig. 4; Reiss and Eisthen 2008). Some fully aquatic anurans such as the pipid *Xenopus laevis* have a sensory epithelium in the lateral diverticulum also known as middle cavity (MC) (Helling 1938; Eisthen 1992). The MC of most other anurans is not lined with sensory epithelium and does not serve an olfactory function (Scalia 1976; Belanger and Corkum 2009). The putative water noses of many other adult anurans are not located in their MC, but rather reside in other parts of the peripheral olfactory organ, e.g., the recessus olfactorius (Fig. 4) and the anterior part of the PC (Helling 1938; Benzekri and Reiss 2012; Jungblut et al. 2020).

The larval main OE mostly consists of a single olfactory surface in the PC, which bears striking morphological similarities to the adult water noses (Fig. 3; Hansen et al. 1998; Syed et al. 2017; Jungblut et al. 2020). In some species that employ a grazing lifestyle, the ventral part of the main OE is lining the roof of the buccal cavity (Benzekri and Reiss 2012; Jungblut et al. 2017). Due to their direct exposure to food contents, these patches of OE could be an adaptation to the specific ecomorphotype of some tadpoles; however, no physiological or molecular data is available on the matter.

Due to the lack of physiological evidence determining the function of air noses and water noses in amphibians, most studies thus far have concentrated on the cellular composition of the various types of epithelia (Fig. 2). Only a few studies have focused on the expression of olfactory receptor proteins that hint towards a function related to water or land-based olfaction and they are limited to very few species.

### Cellular adaptations of the peripheral olfactory organ to different environments

The olfactory mucosae of all vertebrates are basically made of an OE and an adjacent lamina propria (Graziadei 1971; Salazar et al. 2019). All OE, independent of the medium in which they operate (water or air), comprise three main cell types: (i) supporting cells (SCs), (ii) basal cells, and (iii) receptor neurons (Fig. 2; Graziadei 1971; Eisthen and Polese 2007; Taniguchi and Taniguchi 2014). The composition of the lamina propria, on the other hand, partly differs in water noses and air noses. Bowman’s glands are present only in air noses, where they are mainly involved in the production of olfactory mucus (Getchell et al. 1988).

All vertebrate OE (water noses, air noses, and the VNO) comprise characteristic types of SCs (Taniguchi and Taniguchi 2014). These cells form a sort of intraepithelial scaffold that constitutes a largely impermeable barrier (apical tight junctions) between the epithelium and the external world (Steinke et al. 2008; Liang 2020). Generally, SCs have apically located somata and expand basal processes through the epithelium that terminate at the basal lamina (Rafols and Getchell 1983; Chen et al. 2014). SCs carry out a myriad of important functions that go well beyond structural and maintenance tasks. They actively regulate the ionic environment of the OE and the mucus layer (Getchell and Getchell 1992) and they are involved in mucus production (Getchell et al. 1988). Notably, SCs can also influence olfactory signal transduction in receptor neurons, and are generally involved in several epithelial modulatory mechanisms (Lucero 2013). Two major types of SCs are known: ciliated and secretory SCs (Eisthen 1992).

In anuran amphibians, water- and air-type OE as well as the epithelium in the VNO are endowed with characteristic sets of SCs (Fig. 2). While water-type epithelia exhibit both secretory and ciliated SCs, air noses only have secretory and VNOs only ciliated SCs (Hansen et al. 1998; Benzekri and Reiss 2012; Nowack et al. 2013). Secretory SCs are thought to be involved in mucus production similar to goblet cells in fish OEs (Getchell and Getchell 1992) and ciliated SCs may be important for fluid movement across the surface of the epithelium (Eisthen 1992). The distribution of the two SC types is however not uniform among amphibians. In some salamander species, both the main and the accessory OS are endowed with both types of SCs, while other salamanders and the single examined caecilian species were shown to lack ciliated SCs (Jones et al. 1994; Różański and Żuwała 2019). A clear functional association of the various SC types with aquatic, aerial or vomeronasal olfaction can thus not be clearly stated for all extant amphibians and awaits functional verification.

Basal cells are the stem cells of the vertebrate peripheral OS (Fig. 2; Graziadei 1971; Brann and Firestein 2014). Receptor neurons of both, the main OE and the epithelium of the VNO (Brann and Firestein 2014) are periodically replaced by new neurons. There is evidence that also SCs (Schwob and Jang 2006; Leung et al. 2007) and cells of Bowman’s glands (Schwob and Jang 2006) can be substituted from the pool of basal cells. Basal cells have been found in all vertebrates investigated so far (Hassenklöver et al. 2009; Taniguchi and Taniguchi 2014). This supports the hypothesis that all olfactory epithelia are equipped with a local stem cell pool to replenish diminishing receptor neurons independently of the surrounding medium (air or water).

Vertebrate receptor neurons typically have a bipolar morphology, an apical dendrite that ends in a characteristic knob-like structure that bears either cilia or microvilli, and extend an axon towards the OB (Schild and Restrepo 1998; Ache and Young 2005). Generally, the cilia are non-motile (Falk et al. 2015), but in some non-mammalian species, motile olfactory cilia have been described (Lidow and Menco 1984). Olfactory receptor proteins, the binding sites of odor molecules, are located on these apical appendages (cilia and microvilli) (Glezer and Malnic 2019). So far, five different families of vertebrate olfactory receptor proteins, all of them being G-protein coupled receptors (GPCRs), have been identified. These are olfactory receptors proper (OR-type), vomeronasal receptors of type 1 and type 2 (V1Rs and V2Rs), trace amine-associated receptors (TAARs), and formyl peptide receptors (FPRs; so far only confirmed in rodents) (Buck and Axel 1991; Dulac and Axel 1995; Herrada and Dulac 1997; Matsunami and Buck 1997; Ryba and Tirindelli 1997; Liberles and Buck 2006; Rivière et al. 2009; Liberles et al. 2009). A sixth family of proteins serving as olfactory receptors that are not coupled to G-proteins, the so-called membrane-spanning 4A receptors (MS4As), have recently been shown to be co-expressed with guanylate cyclase-D (GC-D) in a third morphological type of receptor neuron, so far only found in the rodent main OE (Greer et al. 2016).

Vertebrate receptor neurons possess two different main transduction cascades, a cyclic monophosphate (cAMP)-dependent one expressed in ciliated receptor neurons and a phospholipase C (PLC)-dependent one in microvillar receptor neurons (Munger et al. 2009; Manzini and Korsching 2011; Bear et al. 2016; Spehr 2017; Mohrhardt et al. 2018). While the cAMP-dependent cascade is coupled to OR-type olfactory receptors and TAARs and relies on the G-protein Gα_olf_, the PLC-dependent one is coupled to vomeronasal receptors and FPRs (Munger et al. 2009; Bear et al. 2016; Mohrhardt et al. 2018) and relies on different G-proteins (mainly Gα_i_ and Gα_o_). These two pathways show different degrees of anatomical segregation in the various vertebrate lineages and olfactory relay stations. In mammals, the cAMP-dependent and the PLC-dependent transduction mechanisms are partitioned between the main OE and the VNO, respectively (Munger et al. 2009). Contrastingly, in most fishes, the two transduction cascades coexist in their singular OE (Olivares and Schmachtenberg 2019).

The most common configuration of the olfactory periphery in amphibians (both larval and adult) consists of microvillous receptor neurons arranged in the VNO and both microvillous as well as ciliated receptor neurons in the main OE (Figs. 3 and 4; Eisthen 1992; Saint Girons and Zylberberg 1992; Eisthen et al. 1994; Hansen et al. 1998; Manzini and Schild 2010; Różański and Żuwała 2019). In the pipid frog *Xenopus laevis*, the microvillar receptor neurons in the larval and adult VNO express V2Rs (Hagino-Yamagishi et al. 2004; Syed et al. 2013) and rely on Gα_o_ and TRPC2 for signal transduction similar to the salamander and mammal VNO (Gliem et al. 2013; Sansone et al. 2014; Kiemnec-Tyburczy et al. 2012). However, Gα_olf_ expressing receptor neurons have additionally been found in the VNO of adult *Bufo japonicus* and the red legged salamander (Hagino-Yamagishi and Nakazawa 2011; Nakada et al. 2014; see also Jungblut et al. 2020). So far, sulfated steroids are the only odor molecules that have been shown to stimulate vomeronasal receptor neurons in larval anurans (Sansone et al. 2015).

In adult anurans, the air-type portion of the main OE in the PC consists of only ciliated receptor neurons (Fig. 4; Föske 1934; Bloom 1954; Menco 1980; Mair et al. 1982; Eisthen 1992; Hansen et al. 1998). Receptor neurons in the adult PC epithelium in *Xenopus* generally express Gα_olf_ (Nakada et al. 2014) and OR-type genes related to the mammalian class II olfactory receptors have been found (Freitag et al. 1995). In rodents, these receptors are responsive to hydrophobic, small volatile chemicals like aldehydes, alcohols or ketones (Saito et al. 2009). Similarly, OR-type receptors have been found to be distributed in distinct zones in the main OE of the tiger salamander (Marchand et al. 2004), putatively responding to small volatile odorants (Kauer 2002). However, also some members of the V1R family were found to be expressed in the adult PC of *Xenopus* (Date-Ito et al. 2008). The expression of TAARs in the adult PC has not been verified yet and the V2Rs expressed in the larval PC progressively vanish from the PC during development (Syed et al. 2017). Especially in the aquatic *Xenopus*, the question whether the “air-system” is biologically relevant is poorly addressed so far. Out of the water, they have been observed in the wild to search for new ponds and lakes thus putatively using their sense of smell for orientation (Kramer 1933; Du Plessis 1966; Measey 2016).

In the various water-type epithelia of anurans (both in tadpoles and adults), both types of receptor neurons are intermingled (Figs. 3 and 4; Hansen et al. 1998; Benzekri and Reiss 2012; Nowack et al. 2013). In the main OS (PC) of larval *Xenopus laevis*, there are two well-described parallel olfactory processing streams with underlying differences in cellular machinery (Manzini and Schild 2010). On the level of the main OE, these streams are only partially segregated, but they are almost fully segregated only in the OB (see below). One stream, likely made of ciliated receptor neurons expressing OR-type olfactory receptors (or possibly TAARs), relies on Gα_olf_ and the cAMP-dependent intracellular signaling pathway and is mostly tuned to detect aldehydes, ketones, and alcohols (Gliem et al. 2013). The second stream, on the other hand, is presumably linked to microvillar receptor neurons with vomeronasal receptor expression, relies on Gα_o_/Gα_i_ and the PLC-dependent intracellular signaling pathway and is tuned to amino acid odorants (Manzini et al. 2002; Manzini and Schild 2003; Gliem et al. 2013). In stark contrast to mammals, in *Xenopus laevis*, an ancient clade of V2Rs and the whole V1R-family are exclusively expressed in the larval main OE (Fig. 3; Date-Ito et al. 2008; Syed et al. 2013; Gliem et al. 2013; Sansone et al. 2014). The water nose of postmetamorphic *Xenopus* (MC) seems to be a molecular copy of the PC found in larvae, with a similar set of receptor genes, transduction elements and odorant sensitivities recorded (Fig. 4; Syed et al. 2017). In the absence of molecular data for the water noses of other species (e.g., recessus olfactorius), it remains unclear whether their molecular setup is comparable to the water noses in *Xenopus* (Fig. 4).

### The olfactory bulb, the first relay station of the olfactory pathway

In vertebrates the axons of receptor neurons break through the basal lamina of the OE, coalesce to form the olfactory nerve, and terminate in so-called glomeruli in the OB, the first relay center of the OS (Nagayama et al. 2014). In mammals, birds, reptiles, and amphibians the OB is made of six more or less discernible layers: (i) the olfactory nerve layer, (ii) the glomerular layer, (iii) the external plexiform layer, (iv) the mitral cell layer, (v) the internal plexiform layer, and (vi) the granule cell layer (Shepherd 1972; Taniguchi and Taniguchi 2014; Nagayama et al. 2014). In fishes, on the other hand, solely four clearly discernable OB layers exist (Olivares and Schmachtenberg 2019). In the OB, the olfactory information is substantially processed before it is relayed to higher olfactory structures, the olfactory cortices (Cleland and Linster 2019).

Within glomeruli, the axons of receptor neurons form excitatory synapses with projection neurons, i.e., mitral cells and tufted cells, and so-called juxtaglomerular cells (Munger et al. 2009; Crespo et al. 2019). The main types of juxtaglomerular cells are: (i) periglomerular cells, (ii) short-axon cells, and (iii) external tufted cells (Nagayama et al. 2014; Kosaka and Kosaka 2016). Within the glomerular modules, a first main processing step of the olfactory information takes place. A second processing step happens in deeper layers of the OB, mainly in the external plexiform layer and projection neuron layers. There, mitral cells and tufted cells mainly form characteristic interactions with granule cells, at so-called dendro-dendritic synapses (Shepherd et al. 2007; Nagayama et al. 2014). The signal can be modulated further by other intrinsic bulbar systems, e.g., an endocannabinoid and a purinergic system (Harvey and Heinbockel 2018; Rotermund et al. 2019). Several extrinsic neuromodulatory projections terminate in the OB and additionally modulate the bulbar output of projection neurons (Rothermel and Wachowiak 2014; Harvey and Heinbockel 2018). Like receptor neurons in the OE, also bulbar interneurons are constantly replaced by newly formed neurons that arise from a pool of neuronal stem cells located in the walls of the lateral cerebral ventricles (Lledo et al. 2005; Takahashi et al. 2018).

While most of the available information about the vertebrate OB comes from studies in rodents, it is generally assumed and very likely that the basic mode of functioning is very similar in most vertebrates. The amphibian OB shares many organizational features with other vertebrate classes, and these do not change substantially during development (Byrd and Burd 1991; Nezlin et al. 2003). Subtle differences from the mammalian OB include missing glial borders around the glomerular neuropil and a more scattered occurrence of projection neurons in non-distinct layers (Scalia et al. 1991a; Byrd and Burd 1991; Kratskin et al. 2000; Rössler et al. 2002; Nezlin et al. 2003).

The OB of species with more than one peripheral OE, i.e., with more than one olfactory subsystem, is divided in subsystem-specific compartments that processes solely information of that specific subsystem (Munger et al. 2009). In both larval and adult amphibians, the VNO and the main OE generally project to the main and accessory OB, respectively (Figs. 3 and 4; Schmidt et al. 1988; Schmidt and Wake 1990; Manzini and Schild 2010; Jungblut et al. 2012). Exceptionally, receptor neurons in the ventral main OE of the fire belly newt *Cynops pyrrhogaster* also project to glomeruli in the accessory OB (Nakada et al. 2014). Apart from the dichotomy between the main and accessory OS, the glomerular projections in caecilians and urodeles have so far not been further subdivided into discernable glomerular clusters (Schmidt et al. 1988; Schmidt and Wake 1990). However, it was shown, that the ventral and dorsal portions of the main OE in the fire belly newt differentially project to different parts of the glomerular array in the main OB, hinting towards a further compartmentalization (Nakada et al. 2014).

In anurans, the most detailed account on the structural organization of glomeruli is provided for larvae from the genus *Xenopus* (Nezlin et al. 2003; Gaudin and Gascuel 2005; Manzini et al. 2007a, 2007b). Generally, the axons of receptor neurons terminate in four clearly segregated glomerular clusters in the main OB (Fig. 3; Weiss et al. 2020a) that can further be subdivided (Gaudin and Gascuel 2005). The PLC-dependent processing stream is mainly amino acid-sensitive and supposedly relies on V1Rs, V2Rs (and possibly TAARs), and Gα_i_/ Gα_o_. The cAMP-dependent stream is sensitive to alcohols, amines and ketones and probably relies on OR-type olfactory receptors (and possibly TAARs) and Gα_olf_. The two streams are only partially segregated in the olfactory periphery (see above), but are almost fully segregated at the level of the main OB (Fig. 3; Manzini and Schild 2010; Syed et al. 2013; Gliem et al. 2013; Bear et al. 2016). While axons of the PLC-dependent receptor neurons terminate almost exclusively in the lateral glomerular cluster, axons of the cAMP-dependent receptor neurons mostly terminate in the medial and dorsal glomerular clusters (Gliem et al. 2013). These two streams are distinguishable in tadpoles, but it remains to be shown how the projections are shaped after the metamorphic climax. It is plausible that at least the coarse functional organization of the ventral main OB and its putative behavioral output remains unaffected by the high amount of cell death and the system-restructuring happening during metamorphosis (Fig. 4; Dittrich et al. 2016; Syed et al. 2017). Intriguingly, an amino-acid sensitive lateral stream was found in the OS of most fishes and was shown to be related to feeding behavior in the carp (Hamdani 2001). A feeding-related behavioral effect of amino acids in *Xenopus* is still not clear (Terni et al. 2017).

A recent study in six distantly related anuran species of four families (Pipidae, Hylidae, Bufonidae, Dendrobatidae) suggests that the glomerular organization of anuran larvae is highly conserved (Weiss et al. 2020a). Major neuropilic clusters in the main OB that were originally identified in *Xenopus* (Gaudin and Gascuel 2005; Manzini et al. 2007a) are also present in the surveyed species, indicating strong developmental constraints despite ecological diversity. Although the general organization is similar, volume differences between the glomerular clusters hint towards a functional emphasis or reduction on certain parts of the olfactory network (Weiss et al. 2020a). Whether these volume differences could be related to the presence or absence of projections originating in the ventral/buccal exposed epithelium (also see Jungblut et al. 2020) remains completely unexplored so far (Fig. 3).

Despite the enormous number of anatomical accounts of amphibian noses, little attention has been paid to the neuronal circuits involved in the respective water and air systems of adult amphibians. The main OB of adult anurans is usually described as a laminar neuronal structure fused at the interhemispheric midline (Herrick 1910; Scalia et al. 1991a; Leveteau et al. 1992; Jiang and Holley 1992; Eisthen and Polese 2007). In most studies, no distinction between projection targets originating in the water or air noses has been made. This could possibly be due to a relatively small size of the water-nose in comparison to the air nose in terrestrial anurans and consequently a neglect to focus on the glomerular portions innervated by the water nose. *Xenopus laevis* is an aquatic frog equipped with a substantial water nose in the MC, but putatively terrestrial ancestors (Wells 2007a; Reiss and Eisthen 2008). The presence of a well-developed aerial OS implies that the adult *Xenopus* is well adapted also to smell on land (Föske 1934). During metamorphic remodeling, newly generated receptor neurons residing in the PC epithelium no longer project to the glomeruli in the ventral main OB, but to a dorso-medial target region (Reiss and Burd 1997; Gaudin and Gascuel 2005). While during premetamorphic stages receptor neurons axons solely project to the glomerular clusters in the ventral main OB, the de novo formed dorsal main OB grows extensively until the end of metamorphosis (Fig. 4; Gaudin and Gascuel 2005).

Apart from the size differences, the dorsal “air-bulb” differs significantly from the ventral main OB in its wiring properties. It has been occasionally noted, that some receptor neuron axons projecting to the dorsal main OB of *Xenopus* innervate contralateral glomeruli (Ebbesson et al. 1986; Leveteau et al. 1992; Reiss and Burd 1997). It is known from different studies using soybean agglutinin, that receptor neurons of the adult *Pipa* and *Xenopus* MC project to ventrally located glomeruli of the main OB (Key and Giorgi 1986; Franceschini et al. 1992; Meyer et al. 1996). The glomeruli of the ventral main OB after metamorphosis closely resemble the glomerular organization in the premetamorphic tadpole of *Xenopus laevis* (Figs. 3 and 4; Gaudin and Gascuel 2005). It remains to be shown if the functional odor map described in previous work by our group (Manzini et al. 2002; Manzini and Schild 2010; Gliem et al. 2013) is retained in glomeruli of the “water-bulb” after metamorphosis.

Recent results from *Dendrobates tinctorius* could help understand whether the ventral main OB projections might be habitat dependent in adult anurans (Weiss et al. 2020a). It is known from dendrobatids that airborne olfaction plays an important role in orientation and homing behavior (Forester and Wisnieski 1991), but clear use of waterborne olfactory cues have not been described. While the glomerular clusters in the ventral main OB in *Xenopus tropicalis* remain morphologically intact during metamorphosis, the ventral main OB of postmetamorphic *Dendrobates tinctorius* shows signs of degeneration or vestigialization in comparison to larval animals (Weiss et al. 2020a). Even though the lateral glomerular cluster remains visible, the medial and intermediate glomerular clusters have almost disappeared. The most plausible reason for this degeneration could be the absence of the recessus olfactorius (see Jungblut et al. 2020), as described in *Dendrobates tinctorius* by Helling (1938). More comparative data will be needed to understand how the ventral “water-bulb” and its function are correlated to the presence or absence of the respective water-epithelia. It is tempting to assume that axonal projections originating in the recessus olfactorius target the same glomeruli in the ventral main OB that are innervated by the larval PC or the MC in *Xenopus* (Fig. 4). However, the projections targets of the recessus have not been described yet.

### Glomerular wiring patterns

**Fig. 5:**
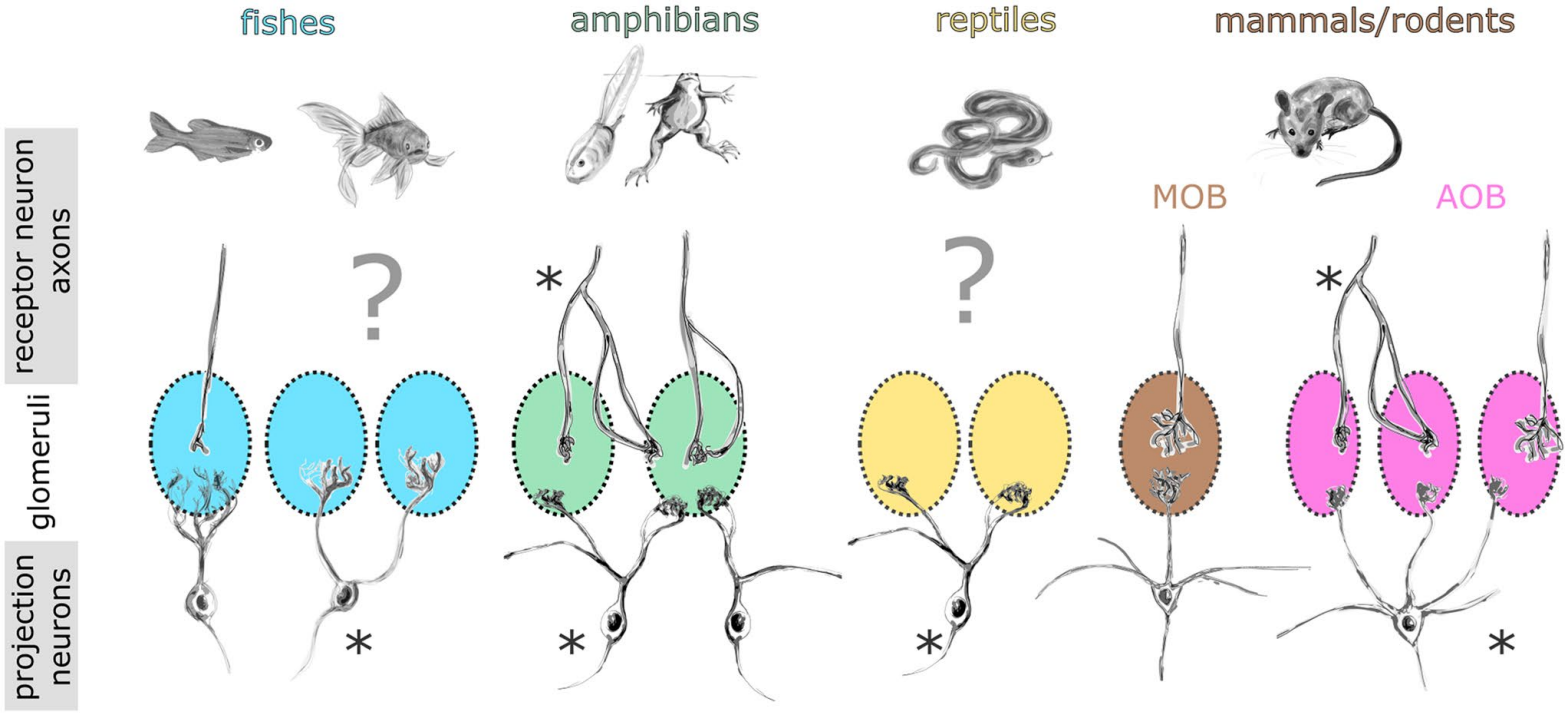
Comparison of glomerular wiring properties in the olfactory bulb of vertebrates. Morphology and glomerular connectivity of single receptor neuron axons and projection neurons are shown. Occurrence of connections to multiple glomeruli are highlighted with an asterisk. In zebrafish (left), both receptor neuron axons and projection neuron dendrites show a uni-glomerular connectivity. Other fishes have multi-glomerular projection neurons, while the morphology of the receptor neuron axons is still unknown. The predominant morphological type of both receptor neurons and projection neurons in amphibians (both main and accessory olfactory system) are multi-glomerular, but uni-glomerular connectivity is also described. Reptiles similarly have multi-glomerular projection neurons; the morphology of the receptor neuron axons is still unknown. In rodents, uni-glomerular connectivity is present in the main olfactory bulb (MOB), while the accessory olfactory bulb (AOB) generally exhibits multi-glomerular projection neurons and only occasional multi-glomerular receptor neuron axons. AOB accessory olfactory bulb, MOB main olfactory bulb

The formation of a glomerular map through axonal projections to the OB relies heavily on the expression of olfactory receptors expressed in the dendrites, but also the axons of receptor neurons (Barnea 2004; Mombaerts 2006). The central dogma in vertebrate olfaction concluded from experiments in rodents says that an individual receptor neuron (both in the main OE and the VNO) expresses a single allele of a single gene from one of the olfactory receptor gene families (Chess et al. 1994; Rodriguez et al. 1999; Serizawa 2003; Shykind et al. 2004). Axonal projections of all receptor neurons expressing the same allele converge onto specific glomeruli (Ressler et al. 1994; Vassar et al. 1994; Mombaerts et al. 1996; Belluscio et al. 1999; Rodriguez et al. 1999). Individual receptor neuron axons in the main OB were shown to be unbranched prior to entering a single glomerulus (Klenoff and Greer 1998). This is true for the OS of rodents, zebrafish, and lamprey (Fig. 5; Klenoff and Greer 1998; Weiss et al. 2020b). Interestingly, in the rat accessory OB, up to 20% of receptor neuron axons bifurcate before entering a single or multiple glomerular structures (Larriva-Sahd 2008). Contrastingly, in the *Xenopus laevis* main OB and accessory OB, receptor neurons exhibit mostly a bifurcating axonal growth pattern with a frequently occurring multi-glomerular innervation (Fig. 5; Nezlin and Schild 2005; Hassenklöver and Manzini 2013). On the presynaptic side, only 30% of receptor neuron axons in the *Xenopus tropicalis* tadpole main OB project to a single glomerulus, while the remaining 70% innervate at least two glomeruli (Weiss et al. 2020b). This ratio is comparable in neobatrachians with differing lifestyles, namely *Rhinella arenarum*, *Scinax granulatus*, and *Ranitomeya imitator* (Weiss et al. 2020b).

The cellular and molecular identity of receptor neurons does not determine if the associated axon connects to single or multiple glomeruli as all patterns were observed both in olfactory organs populated with only ciliated and only microvillous receptor neurons, the adult main OE and the VNO (Jungblut et al. 2009, 2017; Hassenklöver and Manzini 2013; Weiss et al. 2020a, 2020b). These deviations from the “standard” wiring model persist throughout larval development and are retained after metamorphosis (Hassenklöver and Manzini 2013; Weiss et al. 2020b). Axonal projection patterns with multiple connected glomeruli seem to be predominant in anurans, maybe even in amphibians as a whole, since it was also demonstrated in the Axolotl salamander (Weiss et al. 2020b). It is still unclear if similar axonal projection patterns are present also in other species, what advantages this organization provides for the OS and how it is connected to the different types of air–water interfaces that amphibians have to face, especially evolutionarily (Fig. 5).

On the postsynaptic side of the OB circuit, the projection neurons of the amphibian OB feature multiple apical dendrites (Imamura et al. 2020). This is strikingly different from the projection neurons in the mammalian main OS, but has also been described in fishes and reptiles as well as the accessory OB of mammals (Dryer and Graziadei 1994). In amphibians, these dendrites frequently connect to more than one glomerulus (Jiang and Holley 1992; Dryer and Graziadei 1994; Nezlin et al. 2003; Hawkins et al. 2017). Notably, the ratio of single to multi-glomerular connectivity is comparable to the ratio on the presynaptic side, as shown in multiple anuran species (Jiang and Holley 1992; Weiss et al. 2020b). It is thus likely that some fishes and reptiles also have bifurcating receptor neuron axons (Fig. 5).

The convergence ratio of receptor neuron to projection neurons in the OB in anurans is very low in comparison to, e.g., fish and mammals (Byrd and Burd 1991). In *Xenopus laevis*, a ratio of 5:1 in tadpoles and 34:1 in adult frogs has been estimated (Byrd and Burd 1991). It is conceivable that the limited number of receptor neurons converging on single projection neurons is either consequence or necessity of neurite bifurcations in the anuran OS. Based on the presented numbers in anurans, it is tempting to assume that uni-glomerular receptor neuron axons and uni-tufted projection neurons form a connectivity pattern similar to the main OS of rodents, while the multi-glomerular pattern is reminiscent of the rodent accessory OB (Del Punta et al. 2002; Wagner et al. 2006). It is not clear, whether the uni-glomerular and multi-glomerular channels are separate from each other, or whether a single glomerulus can be innervated by a multi-glomerular receptor neuron axon branch and a non-bifurcating axon. Individual projection neurons might receive information from the same olfactory receptor on their multiple dendritic tufts (homotypic input) or integrate between different glomerular inputs (heterotypic input). Support for the former was given by Del Punta et al. (2002), while Wagner et al. (2006) showed that the input was not necessarily from the same receptor allele, but from closely related receptors (selective heterotypic). This could facilitate coincidence detection of several chemicals or odorant blends. However, since these studies were conducted in the rodent AOB, it is difficult to say how much information they hold on the anuran OS.

Further studies will be necessary to understand, if the multi-glomerular pattern occurs contextually in receptor neurons expressing different receptors from putatively different receptor gene families. This will also help to understand, if the underlying odor processing is identical to the rodent accessory OB, or if the similarities are just coincidental.

### Higher olfactory centers, the terminal olfactory processing centers

Our knowledge about how odors are identified and differentiated in the terminal olfactory processing centers, i.e., the olfactory cortices, is still limited and most available information comes from the mammalian system. In mammals, the axons of bulbar projection neurons terminate in one of the several higher and highly interconnected brain areas (Haberly 2001; Klingler 2017) that together are named olfactory cortex. These regions include (i) the anterior olfactory nucleus, (ii) the olfactory tubercle, (iii) the piriform cortex, (iv) the olfactory amygdala, and (v) the entorhinal cortex (Canavan et al. 2011; Cleland and Linster 2019). Differently than in the other sensory systems the olfactory information on its way to the olfactory cortices bypasses the thalamus (Shipley and Ennis 1996). In non-mammalian vertebrate species even less is known about cortical olfactory information processing (Illig and Wilson 2009).

In anurans, the OB projections are divided into lateral and medial olfactory tracts targeting different regions within the pallium (Northcutt and Royce 1975; Scalia 1976). Olfactory projections of the main and accessory OS form distinct information channels on the level of the lateral pallium (Herrick 1921; Northcutt and Royce 1975). Within the lateral pallium, the amygdaloid complex is an important target region for olfactory inputs and can be subdivided into three components: lateral, medial, and central amygdala (Marín et al. 1998; Moreno and González 2006, 2007; González et al. 2020). Projection neurons of the main and accessory OB mainly connect to the lateral and medial amygdala, respectively (Moreno et al. 2005; González et al. 2020). Interestingly, in the medial amygdala a co-innervation of main and accessory projection neurons is present indicating a convergence and integration of the information of these two systems at this level (Scalia et al. 1991b; Moreno and González 2003). It is also notable that some extrabulbar fibers originating from the OE project to deeper diencephalic brain regions by bypassing the OB (Hofmann and Meyer 1992; Pinelli et al. 2004; D’aniello et al. 2008; Gaudin et al. 2013). Functional evidence about processing of olfactory information in higher olfactory centers is scarce and it is unknown how olfactory information from sensory epithelia for water or air is integrated. The differential projections of water and air noses to different compartments of the OB could also translate into segregated projections from the OB to higher brain centers. Unraveling these circuits could help to link detection of airborne and waterborne odor molecules to relevant behaviors.

## Conclusion

Many of the data concerning the anuran OS, including cellular structure, neuronal circuitry, and function, have been derived from studies on *Xenopus*, a basal anuran. A specialized lifestyle and evolutionary distance among anurans make it difficult to accept its OS and glomerular projections as an “anuran blueprint” and additional comparative verification is necessary. Although the general organization of the anuran OS shares many characteristics with other vertebrates, there are clearly some differences. Nevertheless, ecological specialization can have a high impact on the organization of the OS and it will be interesting to find out how different lifestyles shape it. Therefore, anuran amphibians offer a unique opportunity to investigate the adaptations of the sense of smell to the demands of the different environments air and water at an evolutionary, ontogenetic and lifestyle level. The information flow of olfactory-mediated behavior is composed of a specific odor molecule/blend, a detecting olfactory subsystem with specific molecular machinery (including olfactory receptor types), an olfactory bulb representation with associated neuronal processing and finally higher brain centers involved in generation of specific behavior. Unfortunately, much of this information is only partly available in observations connected to anuran olfaction. It would certainly be helpful if future studies were better structurally and functionally coordinated to understand this information flow as a whole. This could in the end help to understand the olfactory demands and neuronal processes that underlie optimized olfaction across the boundary between water and air.

## Conflict of interest

The authors declare that the research was conducted in the absence of any commercial or financial relationships that could be construed as a potential conflict of interest.
